# Cerebrolysin induces hair repigmentation associated to MART-1/Melan-A reactivation

**DOI:** 10.1186/s40001-022-00889-4

**Published:** 2022-11-21

**Authors:** Gustavo Villarreal-Reyna, Rodolfo Garza-Morales, Adolfo Soto-Domínguez, Lorena Montañez-Guerrero, Odila Saucedo-Cárdenas, Minerva Gómez-Flores, Jorge Alejandro Ocampo-Garza, José Juan Pérez-Trujillo, Roberto Montes-de-Oca-Luna

**Affiliations:** 1 Service of Neurosurgery, Doctors Hospital, 64620 Monterrey, Nuevo Leon Mexico; 2 Department of Histology, School of Medicine, Universidad Autonoma de Nuevo Leon, 64460 Monterrey, Nuevo Leon Mexico; 3 Service of Dermatology, Hospital Universitario Jose Eleuterio Gonzalez, 64460 Monterrey, Nuevo Leon Mexico

**Keywords:** Hair graying, Melanin, Melanocytes, Cerebrolysin, Repigmentation

## Abstract

Hair graying, a prototypical sign of human aging, is a progressive loss of pigmentation from growing hair shafts caused by disease and as a side effect of medications. Cerebrolysin is a neuropeptide preparation that mimics the effect of endogenous neurotrophic factors. Cerebrolysin has been widely used in neurologic conditions, such as cerebral stroke, Alzheimer’s disease, and dementia, among others. Cerebrolysin treatment has achieved to regain or maintain the cognitive ability of affected patients; however, up to date, there are no reports about the reactivation of hair pigmentation. We describe a previously not described effect occurring on patients receiving Cerebrolysin treatment for neurologic diseases and whether this effect is associated in reactivation of melanocytes and melanin expression. Here, we report five patients (mean age, 70.6 years), who also had age-related hair graying and scalp hair repigmentation during Cerebrolysin treatment. Macroscopic analysis revealed hair repigmentation consisted in diffuse darkening of the scalp hair. Impregnation and immunostaining analysis were performed on scalp biopsies taken before and after Cerebrolysin treatment; the results showed greater melanin and melanocyte marker MART-1/Melan-A staining following Cerebrolysin treatment. We present, to our knowledge, the first report on hair repigmentation is a previously not described effect occurring following Cerebrolysin treatment.

## Introduction

Hair graying is one of the prototypical signs of human aging and occurs in all individuals regardless of sex and race [1]. This process is caused by a progressive loss of pigmentation from growing hair shafts, and it has been associated with several conditions, such as pernicious anemia, hypothyroidism, and osteopenia, among others [2, 3]. Several studies have also reported that hair graying is not an irreversible process, and hair repigmentation has been described the following treatment with medications, such as secukinumab and brentuximab [4–7].

Several studies have provided valuable insight in the possible mechanisms and factors that contribute to hair graying. Hair graying is associated with several processes such as defective self-maintenance, depletion of melanocyte stem cells [8], and increased melanocyte death by apoptosis and oxidative stress [9, 10].

Cerebrolysin is a registered mark (^®^) that is parentally administered, low-molecular weight neuropeptide preparation obtained through standardized enzymatic proteolysis of porcine brain proteins and exhibits neuroprotective and neurotrophic properties similar to those that occur naturally in neurotrophic growth factors [11, 12]. Cerebrolysin has been approved in countries outside of the United States mainly for the treatment of dementia, acute ischemic stroke, cognitive impairment, and Traumatic Brain Injury (TBI) [13, 14].

Numerous studies are currently evaluating the efficacy of Cerebrolysin for other conditions, including Cerebral Palsy [15], vascular dementia [16], and Aneurysmal Subarachnoid Hemorrhage [17]. The pharmacologic effects of Cerebrolysin are of relevance for cerebrovascular and neurodegenerative diseases, as these diseases generate a pathologic environment, which is deleterious for neurons, causing their degeneration, dysfunction, and death either immediately or over time [15, 17]. Experimental studies in stroke models have shown that Cerebrolysin stabilizes the structural integrity of neurons via the inhibition of calpain, supports the formation of neuronal networks by inducing neuronal sprouting and neurogenesis, and enhances the functional recovery accompanied by a reduction in infarct volume [18–20]. A recent meta-analysis demonstrated that this peptide mixture can be used as a safe therapy to recover from severe traumatic brain injuries [21]. Later, a randomized controlled trial in infants with neurodevelopment delay risk showed its benefits as an add-on therapy to reduce incidence of motor and language development issues compared to infants receiving routine intervention only [10 (33%) versus 21 (70%), respectively, *p* = 0.009] [22].

The most frequently reported self-limited adverse reactions due to Cerebrolysin are vertigo or dizziness, headache, nausea, and increased sweating, as stated in the Product Information Label. Some authors also have reported urinary tract infection and fever as adverse reactions, but these were related to the infusion treatment rather than to Cerebrolysin [13, 14]. However, up to date, there are no reports about changes in hair pigmentation related to Cerebrolysin. In the present study, our aim was to describe a previously not described effect observed in patients on Cerebrolysin therapy prescribed by a medical order for neurological diseases and to evaluate whether this effect is associated with an increase in melanin and/or the number of melanocytes on their scalp.

## Methods

### Study design and participants

We developed a prospective, longitudinal, comparative pilot study at the Neurosurgery Service from March 2015 to March 2016. It was conducted in accordance with the Declaration of Helsinki and approved by the ethical committee of School of Medicine, under Protocol no. NC15-001. It is important to state that the present study was performed on patients enrolled in Cerebrolysin treatment for supporting the maintenance and repair of neuronal tissue as a follow-up treatment prescribed by their neurologist; hence, this study was not a clinical trial for recruiting or dose adjusting for repigmentation purposes. Written informed consent for scalp biopsies collection and photos publication was obtained from participant patients.

Patients older than the age of 40 were recruited if they met the study inclusion criteria of the following: (i) known neurologist prescription for Cerebrolysin treatment as a maintenance follow-up due to ischemic or hemorrhagic stroke, traumatic brain injury or dementia, and (ii) age-related white or gray hair. Hair whitening score (HWS) was used to evaluate the macroscopic gray/white hairs proportion on patient scalp and categorized as follows: 1: pure black; 2: black > white; 3: black = white; 4: white > black; and 5: pure white [23]. Cerebrolysin is contraindicated for patients with diagnosis of Chronic Kidney Disease (CKD), a known history of Seizures, Epilepsy, or Hypersensitivity to one or any drug components; therefore, in our study we did not have any of these cases, as also pregnant patients. We excluded patients with history of recent treatment of hair coloring products. Information is registered at ClinicalTrials.gov (Identifier number: NCT05288465).

### Cerebrolysin treatment and scalp biopsy on patients.

The patients who qualified for study entry received the study medication using a modified Cerebrolysin protocol as follows. The treatment consisted of intravenous (iv) infusions of 5 vials per week, 2 vials on Mondays, and 3 vials on Thursdays for 4 weeks, followed by an 8-week resting period prior to the next cycle of treatment. The previous was designed by the participating neurosurgeons in order to reduce the number of interventions in the patients and to improve their adherence to the therapy. This scheme fits the safety doses recommended in Cerebrolysin information label. Each patient had 3 cycles giving a total of 9 months of follow-up. The intravenous infusion was prepared as follows, each 10 mL vial containing 215.2 mg/mL of Cerebrolysin (EVER Pharma, Austria) was diluted with physiological saline solution (NaCI 0.9%) to a final volume of 100 mL, and the infusion duration was approximately 60 min. All infusions were administered in the clinical facility at approximately the same time day. All patients were treated according to the standard care protocols.

Two biopsies were performed in each patient: one initial biopsy in the achromotrichia area on the scalp before the treatment and the other in the repigmented hair area on the scalp after the treatment. Antisepsis of the area was performed prior to each biopsy with chlorhexidine, and the procedure was made under local anesthesia with lidocaine 2% and epinephrine. A 4-mm punch biopsy was used and the wound was sutured with non-absorbable stitches with Prolene 4-0, which were removed in 10 days.

### Morphological analysis

Formalin-fixed, paraffin-embedded scalp tissue samples from participants (*n* = 5) were processed for histopathological, histochemical, and immunolabeling analysis.

For histopathological and histochemical evaluation, paraffin sections (5 μm) were stained with hematoxylin and eosin (H and E) to evaluate the histological characteristics or Fontana-Masson’s histochemical staining to identify melanin. Digital high-resolution images were obtained with a Nikon Microscope Eclipse 50i with an image analysis system on NIS-elements software (Digital Sight dDS-2Mu). Eight random fields per patient at pre- and post-treatment were chosen from Fontana-Masson’s sections. These were analyzed with a high power (40×) objective to quantify the positivity of melanin with the software Image J version 1.51 (National Institutes of Health). The average value and standard deviation (SD) were calculated for morphometric data.

For Immunohistochemical labeling, 5-µm tissue sections were rehydrated through dilutions from absolute ethanol to water, processed in citrate buffer pH 6.0 for antigen retrieval, and blocked with 5% inactivated horse serum in phosphate-buffered saline (PBS) 1 × for 30 min at 37 °C. Labeling was performed using a mouse monoclonal antibody against Melan-A (Cat#A103, Santa Cruz Biotechnology, Dallas, TX, USA) at 1:50 dilution followed by the Labeled Streptavidin–biotin (LSAB) Immunohistochemistry Protocol (VitroVivo Biotechnology, Rockville, MD, USA). Samples were evaluated by light microscopy and digitalized as previously described.

### Statistical analysis

For analysis of morphometric data, results were statistically analyzed with GraphPad Prism 8.0 using the t-Student test; a value of *p* ≤ 0.01 was significant.

## Results

Macroscopic analysis of the effect of Cerebrolysin on hair pigmentation

**Fig. 1:**
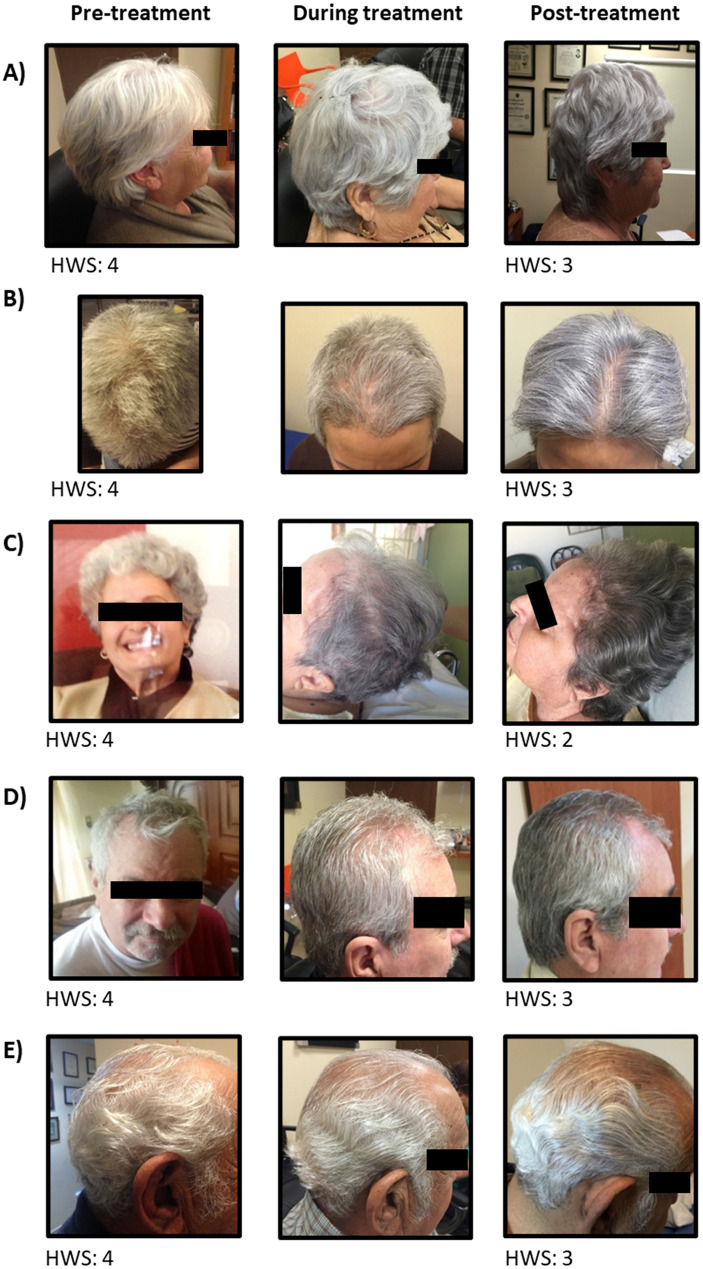
Cerebrolysin-induced hair repigmentation on the patient scalp. Hair pigmentation can be macroscopically detected as an increase in the grayish coloration of the hair root. **A**–**E** Representative time-lapse photos of patients treated with Cerebrolysin are shown

We recruited 5 patients (3 males, 2 females; median age 70.6 years, range 63–77 years) who had severe macroscopic signals of age-related hair graying and were enrolled to receive Cerebrolysin treatment as prescribed therapy for their indicated disease. Taking as reference the hair appearance at the beginning of treatment and the gradual development of pigmentation following treatment, we observed that the patient’s hair began to partially repigment, showing a diffuse dark gray color, predominantly in the temporal and occipital regions, which was in marked contrast from the white hair seen before treatment (Fig. 1). This was reported as an improvement on the Hair Whitening Score (HWS) on all recruited patients (Table 1).

**Table 1:** Clinical characteristics of participant patients who received Cerebrolysin treatment

Patient no.	Sex/Age	Indication for therapy	Pre-hair whitening score	Post-hair whitening score
1	F/77	Ischemic stroke and vascular dementia	4	3
2	F/63	Hemorrhagic stroke due to AcoA aneurysm rupture	4	3
3	F/72	Ischemic stroke with hemorrhagic transformation	4	2
4	M/67	Left parietal ischemic stroke	4	3
5	M/74	Dementia	4	3

Clinical response was determined by physician. Hair repigmentation response was obtained by macroscopic evaluation of post-treatment versus pre-treatment hair color records

*AcoA* anterior communicating artery

Cerebrolysin treatment is associated with an increase of melanin in the hair bulb

**Fig. 2:**
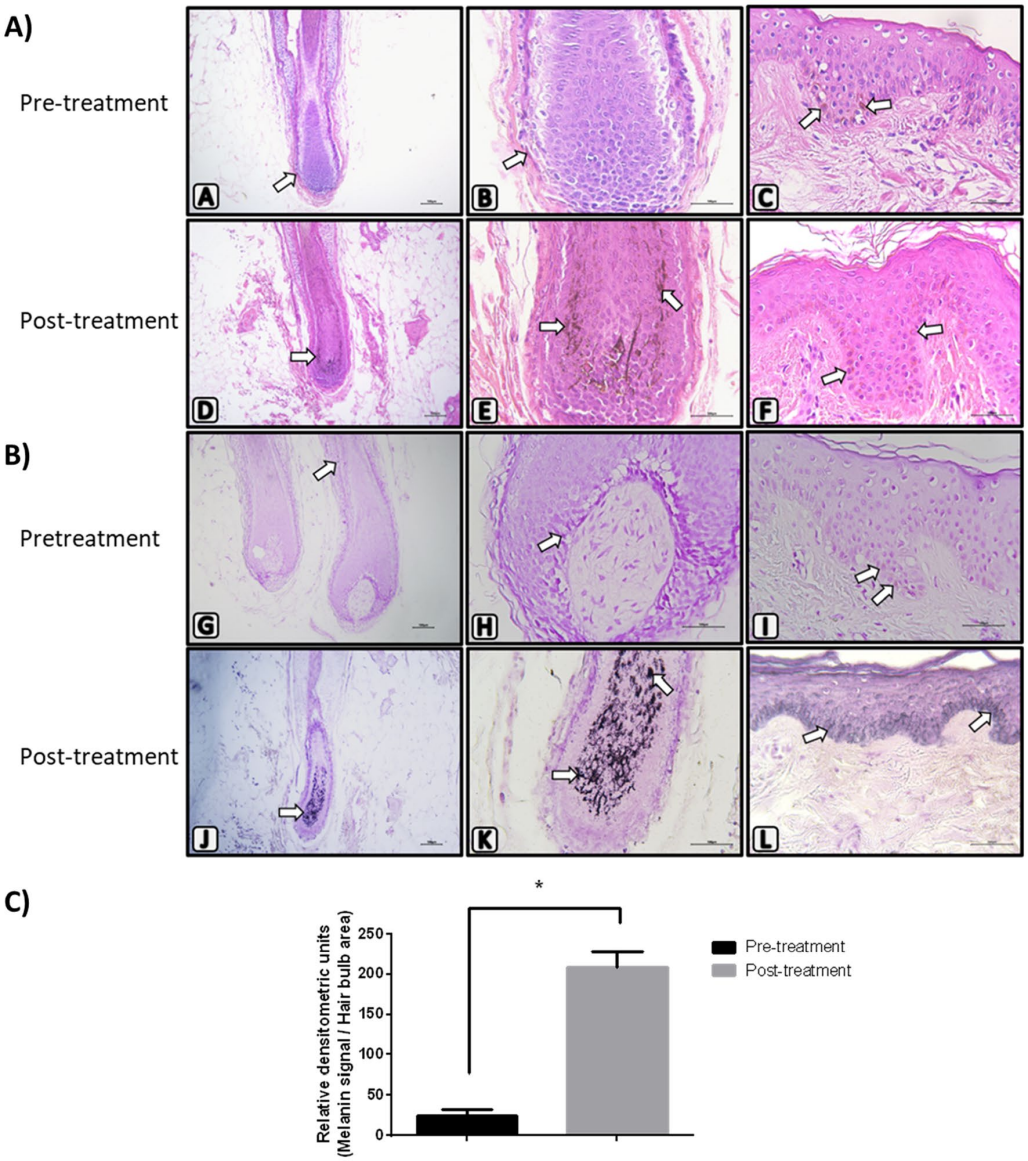
Treatment with Cerebrolysin induces an increment of melanin production. Photomicrographs of pre-treatment (**A**–**C**, **G**–**I**) and post-treatment scalp biopsies (**D**–**F**, **J**–**L**) showing brown-black stain to the melanin granules of melanocytes (white arrows). **A** A–F slides with H and E staining show the brownish melanin color; **B** G–L slides with Fontana-Masson histochemical staining show the black stains due to argentic deposition of melanin. It is noted that post-treatment sections show a greater number and intensity signal in the hair bulb, hair shaft, and epidermis. **A**, **D**, **G**, **J** at 100 × magnification; **B**, **C**, **E**, **F**, **K**, **L** at 400 × magnification; **C** Densitometric quantification of the melanin signal on Fontana-Masson slides. A significant increase (*shows *p* < 0.01) in melanin signal in the hair bulb was detected in post-treatment biopsies. *n* = 3. Experiments were independently performed at least in duplicate

In order to evaluate whether Cerebrolysin treatment has an effect on the amount of melanin on hair follicles, punch biopsies of scalp skin were obtained from patients before and after the Cerebrolysin treatment. Morphological analysis by H and E staining revealed that after receiving Cerebrolysin a greater dark signal due to melanin of melanocytes was observed in the patient scalp and hair bulbs, and the hair shaft also showed greater coloration (Fig. 2A). Hence, to validate the previous, a Fontana-Masson histochemistry was performed to specifically identify melanin by silver impregnation. In Fig. 2B, the results show melanin positivity was evident in both the hair bulb and the skin of the post-Cerebrolysin treatment samples. We detected melanin deposition inside hair follicle (Fig. 2B “K”), as also a greater number of melanocytes with melanin inclusions (Fig. 2B “L”). Moreover, a microdensitometric analysis on Image J revealed a fourfold increase (*p* ≤ 0.01) of melanin staining after the Cerebrolysin treatment (Fig. 2C).

Cerebrolysin treatment associated with melanosome reactivation

**Fig. 3:**
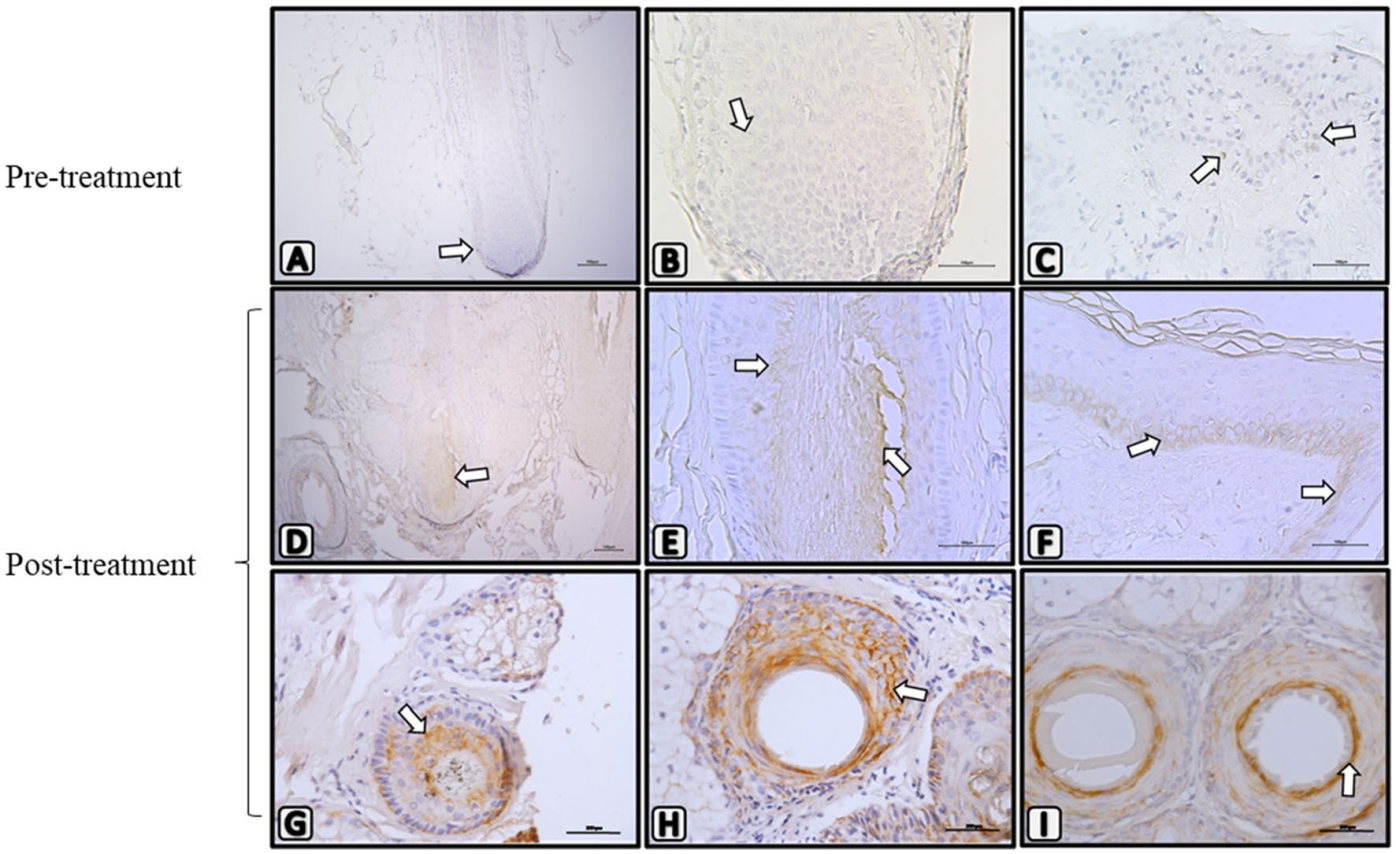
Treatment with Cerebrolysin is associated with an increase in melanocyte marker, Melan-A. Photomicrographs of pre-treatment (**A**–**C**) and post-treatment scalp biopsies (**D**–**I**) showing immunohistochemical staining in the cytoplasm to Melan-A (white arrows). Longitudinal sections of the hair follicle bulge after treatment (**D**–**F**) show an increased positivity when compared to pre-treatment biopsies. Transversal sections of hair follicles shows that positivity is at lower (**G**), middle (**H**), and upper (**I**) bulge regions. (**A**, **D**) 100X Magnification, (**B**, **C**, **E**–**I**) 400X Magnification. *n* = 3. Experiments were independently performed at least in duplicate

Due to the increment of melanin registered at the light microscopy analysis, we proceeded to determine if melanosome reactivation was occurring in hair bulb melanocytes. Therefore, an immunodetection was performed using a mouse monoclonal specific antibody against Melan-A, also known as Melanoma antigen recognized by T-cells (MART-1), responsible for triggering the synthesis of melanin inclusions. The results show positivity in both the hair bulb and the epidermis of post-treatment samples (Fig. 3D–F) showed a greater number of positive cells when compared to pre-treatment samples (Fig. 3A–C). On transversal sections of hair bulbs, we detect that Melan-A positive melanocytes can be located at lower (G), middle (H), and upper (I) bulge regions.

## Discussion

This work represents, to our knowledge, the first evidence of hair repigmentation linked with Cerebrolysin treatment. Hair graying is one of the prototypical signs of human aging, which is caused by a progressive loss of pigmentation from growing hair shafts [1]. The main factor in the tone of skin and hair is melanin, produced by melanocytes, through the melanogenesis pathway. This process occurs regardless of gender or race but the age of graying varies with gender and race [1, 24, 25]. The loss of CD200^+^ stem cells from hair follicles causes a depigmentation in humans linked to chronological graying [26]. These stem cells reside in a niche within the hair follicle, while the differentiated melanocytes reside in the hair follicle bulb [8, 27]. Independent to the stem cells integrity, melanocytes can also be affected on their melanin synthesis signaling and cause a reduction of pigment in hair.

In our study, the reactivation of melanosome, organelle for synthesis and storage of melanin, was evidenced by immunodetection of the melanocyte marker, Melan-A, also known as MART-1, which has been reported to participate in early melanosome biogenesis [28, 29]. This was observed as an increment of melanocytes that had higher Melan-A signal, as also more melanin inclusions, after receiving Cerebrolysin when compared prior to treatment. The latter was observed on macroscopic evaluation as an increase in the proportion of pigmented hairs to white/gray hairs appeal [23]. Although some hairs still have gray tips, there are hair follicle roots that have higher pigmentation than prior to treatment, and this can suggest a recent reactivation in melanin synthesis linked to Cerebrolysin treatment.

To date, there are no reports in the literature regarding the impact of Cerebrolysin on the hair follicle and in particular on the melanocytes. However, it has been determined that Cerebrolysin possess properties of neurotrophic factors [30–32]; these neurotrophic factors are essential components for the microenvironment of stem cells. For example, the RET receptor for neurotrophic factors, which is expressed by stem cells, when activated by these factors boosts their survival, expansion, and function [33]. It should be noted that melanocytes are derived from stem cells present in the hair follicle [25]. In addition, it has been reported that activation of the p75 neurotrophin receptor (p75NTR) is crucial for the regulation of apoptosis in the external root sheath of the hair follicle, in which a niche of stem cells is found [34]. All of the above-mentioned evidence the relationships between neurotrophic factors, stem cells, and hair follicles.

Besides, Cerebrolysin possesses neurotrophic and neuroprotective effects on neuronal survival after different types of lesions in vitro and in animal models of neurodegenerative diseases [11, 35]. The mechanism of action is based on the modulation of four neurobiological processes: neurotrophicity, neuroprotection, neuroplasticity, and neurogenesis [30, 31, 36]. Melanocytes are cells derived from the neural crest during embryonic development; hence, these could be responsive to Cerebrolysin neuropeptide mixture and resulting in the increase of hair pigmentation observed in this group of patients.

To date, the mechanism of activation of melanocytes remains unknown; however, it has been shown that Cerebrolysin may interact with proteolytic pathways linked to apoptosis. Administration of Cerebrolysin significantly reduces the number of apoptotic neurons after glutamate exposure [30], blocking in vitro apoptosis in neurons in addition to increasing cell viability [37]. Another study shows that Cerebrolysin augmented proliferation, differentiation, and migration of subventricular zone neural progenitor cells [38]. Furthermore, Cerebrolysin showed a protective effect on oxidative stress-induced apoptosis in human lymphocytes [30] and neurons [39]; this could also be related to proliferation, migration, and survival of melanocytes against oxidative stress-induced apoptosis that causes hair graying [40].

Moreover, it has been reported that α-melanocyte-stimulating hormone (α-MSH) is decreased in plasma of patients with acute brain injury [41]; it has been shown that nano-delivery of Cerebrolysin and α-MSH induced superior neuroprotection [42]. This may be related to stimulation of the synthesis of melanin by the melanocytes of the treated patients, since studies by Tobin et al*.* also support the hypothesis that follicular and epidermal melanocytes in human skin are different regarding their responses to various biological response modifiers, including α-MSH [43].

Here we report a pilot study of 5 cases of hair repigmentation in patients undergoing Cerebrolysin treatment for diverse medical conditions, and who also suffered of age-related hair graying. We observed that this effect was associated with an increase in melanin and in the melanocyte marker Melan-A. Further studies enrolling more patients and longer treatment therapies are required to gain insight into the mechanisms of Cerebrolysin-induced hair repigmentation.

## Data Availability

Data sharing is not applicable to this article as no new data were created or analyzed in this study.
